# Supplementary figures and images for: Problem-solving therapy for pregnant women experiencing depressive symptoms and intimate partner violence: A randomised, controlled feasibility trial in rural Ethiopia
Source: PLOS Glob Public Health. 2023 Oct 27;3(10):e0002054. doi: 10.1371/journal.pgph.0002054 (PMC10610520; doi:10.1371/journal.pgph.0002054)

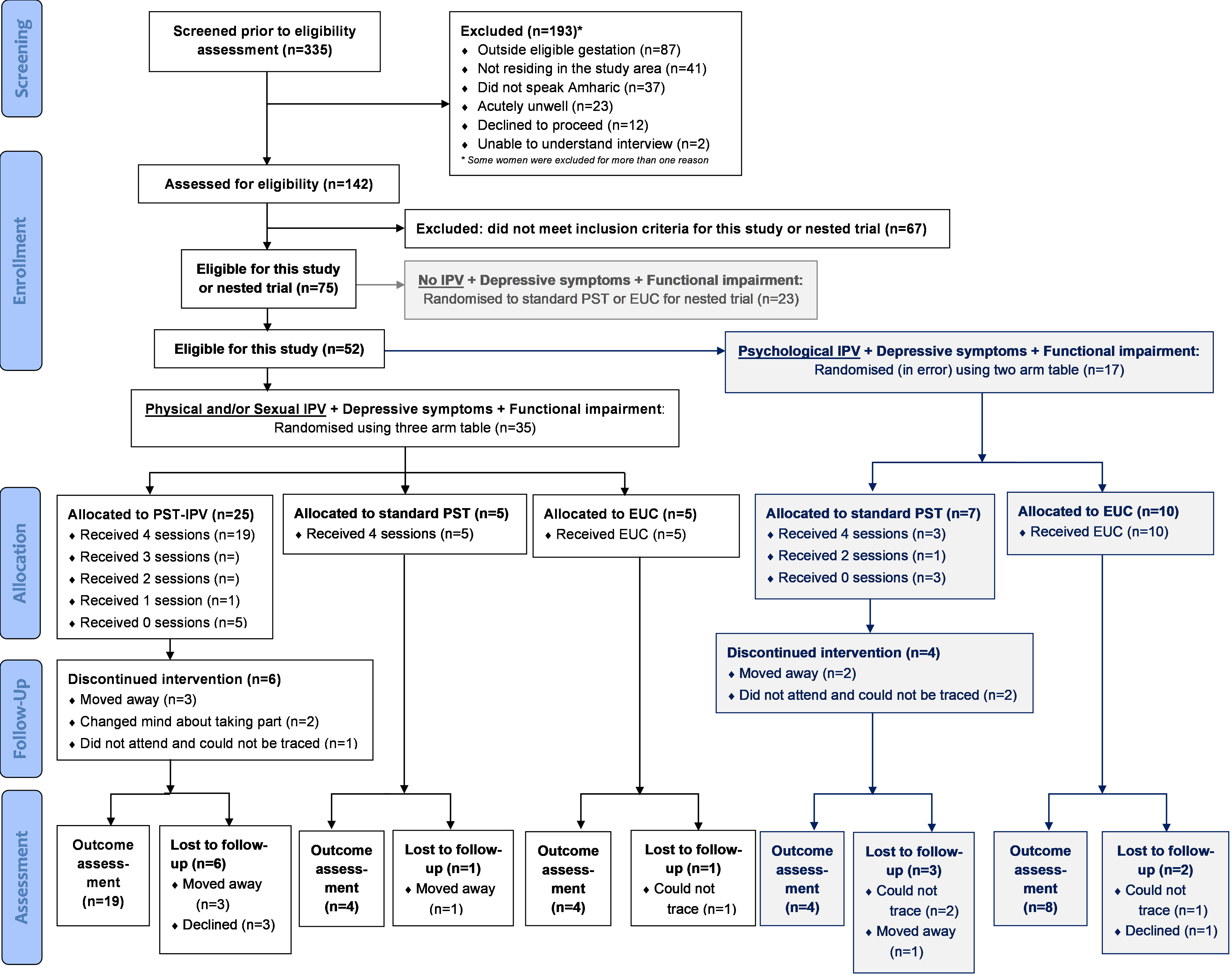

Supplement: S1 Fig — (TIF) [file pgph.0002054.s002.tif]
