# Supplementary material for: Problem-solving therapy for pregnant women experiencing depressive symptoms and intimate partner violence: A randomised, controlled feasibility trial in rural Ethiopia
Source: PLOS Glob Public Health. 2023 Oct 27;3(10):e0002054. doi: 10.1371/journal.pgph.0002054 (PMC10610520; doi:10.1371/journal.pgph.0002054)
Supplement: S1 Table — (DOCX) [file pgph.0002054.s003.docx]

## S1 Table PST-IPV session content

| **Session 1: Welcome to problem-solving therapy (PST)**^[[1]](#footnote-1)^ |
| --- |
| 1. Brief summary of intervention rationale.^[[2]](#footnote-2)^ 2. Starting PST. Brief summary of what to expect. 3. Summary of the overall process of PST. 4. Phase 1: what are the most important things in your life? 5. Guided discussion of the most important things in the woman’s life. 6. Phase 2: What are your worries or problems? Guided discussion of the woman’s worries or problems. Explanation of (A) Lower Priority Problems. 7. Explanation of (B) Problems that Cannot be Changed and (C) Problems that Can be Changed. Guided categorisation of the woman’s worries and problems into different types. Attention paid that any IPV-related problems are listed under (C): Problems that Can be Changed. 8. Phase 3: Planning for the most important things in your life: ‘Problem Busting Session’ 1: applying the 6 step approach to a (C): Problem that Can be Changed. Step 1: name her problem in detail. 9. Step 2: think of as many solutions as you can, Step 3: choose the best approach, Step 4: make a plan. 10. Step 5: take action, Step 6: see if the plan worked, in Session 2. Explanation of what happens if the plan does not work. 11. Summary of Take-Home Activity 1: 12. Put her plan into action and discuss how it went in Session 2. 13. Keep thinking about the most important things in her life. 14. Review her list of problems and worries each day and think about whether they are Lower Priority Problems, Problems that Cannot be Changed or Problems that Can be Changed.   Agree the date and time of Session 2. |
| **Session 2: Coping with different types of problems** |
| 1. Review: of well-being and any progress since Session 1. Summary of the three stages of PST. 2. Recap of the three types of problems and the woman’s examples of each type. 3. Review Take-Home Activity 1: review the most important things in her life and list of problems and worries. Review Problem Busting Session 1. 4. Review progress since Session 1. If her plan did not solve her problem, identifying the next best approach and making a new plan to approach her problem. 5. Coping with Lower Priority Problems: reviewing her Lower Priority Problems and brief explanation about coping. 6. Worry Time: explanation and discussion of how she might use it. 7. Thinking Stop: explanation and discussion of how she might use it. 8. Positive Thoughts: explanation and discussion of how she might use them. 9. Coping with Problems that Can be Changed: Problem Busting Session 2: applying the 6 step approach to a different Problem that Can be Changed. 10. Summary of Take-Home Activity 2: 11. Put her plan(s) for Problems that Can be Changed into action and discuss how they went in Session 3. 12. Put her plans into action for Lower Priority Problems.   Agree the date and time of Session 3. |
| **Session 3: Developing your Problem Solving Skills** |
| 1. Review: of well-being and any progress since Session 2. Summary of the three coping techniques for Lower Priority Problems. 2. Review whether she tried any of these (if not, why not, if so, whether they were helpful, if so, why, if not, why not), and discuss how they could be more helpful. 3. Review Take-Home Activity 2: Review progress made since Session 2 on Problem Busting Session 1 (if a new plan was made in Session 2) and Problem Busting Session 2. 4. Review whether her plan(s) solved her problem(s), identify the next best approach(es) if they did not, and make new plan(s) to approach these problem(s). 5. Coping with Problems that Cannot be Changed: summarise this type of problem and review which problems identified in Session 1 fell into this category. 6. Identify any new Problems that Cannot be Changed which she has identified since Session 1. Summary of loss and coping. 7. The Stages of Acceptance: summary of denial, anger, bargaining, depression. 8. Summary of acceptance. 9. Accepting your feelings and Talking with Others: summary of these coping strategies. 10. Contact with people who share your experience: summary and discussion of how she could use these strategies. 11. Exercises to Reduce Stress 1: Slow Breathing: guided relaxation exercise 12. Continuation of slow breathing exercise. 13. Exercises to Reduce Stress 2: Progressive Muscle Relaxation: guided relaxation exercise. 14. Continuation of progressive muscle relaxation exercise. 15. Coping with Problems that Can be Changed: Problem Busting Session 3: applying the 6 step approach to a different problem that can be changed. 16. Summary of Take-Home Activity 3: 17. Put her plan(s) for Problems that Can be Changed into action and discuss how they went in Session 4. 18. Put her plans into action for Problems that Cannot be Changed. 19. Try the Slow Breathing and Progressive Muscle Relaxation Exercises at home. 20. Try a Problem Busting Session on her own, using the 6 step model.   Agree the date and time of Session 4. |
| **Session 4: Ending Problem-Solving Therapy and using it in Future** |
| 1. Review: of well-being and any progress since Session 3. Summary of the three coping techniques for Problems that Cannot be Changed and the two Exercises to Reduce Stress. 2. Review whether she tried any of these (if not, why not, if so, whether they were helpful, if so, why, if not, why not) and discuss how they could be more helpful. 3. Review Take-Home Activity 3: Review progress made since Session 3 on Problem Busting Sessions 1 and 2 (if new plans were made in Session 3) and Problem Busting Session 3. 4. Review whether her plans solved her problems, identify the next best approaches if they did not and make new plan(s) to approach these problem(s). 5. Review her self-directed 6 Step Approach: review how it went, if she completed this activity. 6. Final Problem Busting Session: for a new Problem that Can be Changed. 7. Using these skills in the future: Summarise what she has learned (Problems that Can be Changed, Lower Priority Problems). 8. Summarise what she has learned (Problems that Cannot be Changed, Exercises to Reduce Stress). 9. Summary and ending: Final summary of how she can apply these techniques to future problems. 10. Discussion about applying these techniques in the future, ways of practising them regularly and who she can talk to if she finds this difficult. |

1. No abbreviations were used in Amharic. [↑](#footnote-ref-1)
2. Numbering refers to pages of the flipchart used to guide intervention sessions. [↑](#footnote-ref-2)
