# Supplementary material for: Problem-solving therapy for pregnant women experiencing depressive symptoms and intimate partner violence: A randomised, controlled feasibility trial in rural Ethiopia
Source: PLOS Glob Public Health. 2023 Oct 27;3(10):e0002054. doi: 10.1371/journal.pgph.0002054 (PMC10610520; doi:10.1371/journal.pgph.0002054)
Supplement: Attachment — Submitted filename: Peer review_Plos Global Health_06.20.23_MS.docx [file pgph.0002054.s004.docx]

**Peer review of**

*Problem-solving therapy for pregnant women experiencing depressive symptoms and intimate partner violence: a randomised, controlled feasibility trial in rural Ethiopia*

**General comments**

This is an excellent study and well-written manuscript. The paper will form an important contribution to the literature after very minor edits, which I outline below.

**Specific feedback**

**Abstract**

Third paragraph: *Fidelity to randomisation was impeded by strong cultural norms about what constituted IPV. However, the trial was feasible (recruitment rate: 1.5 per day; 37% of women screened were eligible).*

Since randomization is a critical component of an RCT, it would be more accurate to state that recruitment (not the trial) was feasible.

Likewise, it would make sense in paragraph four, regarding adjustments, to address the threat to randomization along with the other adjustments listed (e.g., training to identify psychological and emotional IPV).

**Introduction**

Excellent work identifying and justifying the population of focus (POF), the factors (and relationship between factors) affecting the POF’s health, the gaps in the lit, the logical intervention, and the rationale for a feasibility study.

More information is needed, however, to explain why the authors believe a “brief” psychological intervention, as opposed to a more in-depth intervention, is an advantageous choice for this POF and setting. What lit corroborates this choice? What circumstances justify this choice? While I realize this was probably the subject of prior papers, please provide something here as well (including references to those prior, formative studies).

I have only one other suggestion:

First paragraph: *Incident depression was associated with intimate partner violence (IPV) during pregnancy.*

I suggest including the applicable inferential stat estimate here, since OR is consistently reported above and would be informative. Also, please add citation for this sentence.

**Methods**

Figure 1 is excellent. It is very easy to ascertain from the figure and the methods section text that bioethical requirements, including participant safety, were given foremost attention. The flow from recruitment through randomization into study arms is clear.

The methods section provides a very informative level of detail. Missing, however, is whether the participants received compensation or other incentives for their participation.

Under “outcome measurement,” the authors state: *Outcome assessments were performed by masked, independent data collectors.*

It was initially unclear to me if “masked” meant the assessors were blinded to participant assignment to study arm. This was clarified later. To avoid initial confusion, I suggest modifying the text to make this clear.

Again, the methods section is comprehensive and clearly demonstrates the study’s rigor. This level of commitment to methodological detail is often missing in the lit, so I particularly appreciate the effort.

**Results**

The results are comprehensive and articulated will in text and tables. An impressive amount of data were collected, and it is clear that the eventual RCT will likely capture sufficient data to measure direct in indirect effects. Obviously, measuring the statistical significance of pre-post differences was not applicable here. Nonetheless, the authors provide excellent summary tables that allow the reader to see how within and between subjects tests would be executed in a fully-powered RCT. Meanwhile, the study results are important and informative in their own right, addressing gaps the authors identified in the introduction.

I have no changes to suggest here.

**Discussion**

Paragraph three brings up important considerations regarding IPV disclosure. I agree with most of the logic. It would be interesting to stratify by IPV disclosure. However, disclosure would seem to play a powerful role in problem solving, as lack of disclosure is associated with avoidance coping. I am not suggesting changing the text, but it does appear to introduce coping style as a potential confounder.

Overall, the discussion is excellent. I have no suggestions for changes.
