# Supplementary material for: Problem-solving therapy for pregnant women experiencing depressive symptoms and intimate partner violence: A randomised, controlled feasibility trial in rural Ethiopia
Source: PLOS Glob Public Health. 2023 Oct 27;3(10):e0002054. doi: 10.1371/journal.pgph.0002054 (PMC10610520; doi:10.1371/journal.pgph.0002054)
Supplement: Attachment — Submitted filename: Plos Global Health_response_letter.docx [file pgph.0002054.s005.docx]

Section of Women’s Mental Health

Health Service and Population Research department

Institute of Psychiatry Psychology & Neuroscience

King’s College London

PO31 Denmark Hill

London SE5 8AF

[roxanne.1.keynejad@kcl.ac.uk](mailto:Roxanne.1.keynejad@kcl.ac.uk)

Monday 21^st^ August 2023

Dear Dr Waqas,

**RE: Manuscript ID PGPH-D-23-00877 “Problem-solving therapy for pregnant women experiencing depressive symptoms and intimate partner violence: a randomised, controlled feasibility trial in rural Ethiopia”**

Thank you to you and the reviewers for taking the time to consider our article submission to *PLoS Global Public Health*, and for the opportunity to submit a new version. We found the feedback to be helpful and constructive, and believe that our new, revised manuscript has been enhanced by the peer review process.

We have endeavoured to address your and the reviewers’ comments as far as possible, as outlined below. Underlines indicate new text.

Journal Requirements

- Please provide separate figure files in .tif or .eps format

We have done so.

- Please review your reference list to ensure that it is complete and correct. If you have cited papers that have been retracted, please include the rationale for doing so in the manuscript text, or remove these references and replace them with relevant current references. Any changes to the reference list should be mentioned in the rebuttal letter that accompanies your revised manuscript. If you need to cite a retracted article, indicate the article’s retracted status in the References list and also include a citation and full reference for the retraction notice.

We have reviewed the reference list to ensure that all journal titles are similarly capitalised. We have amended the reference for Keynejad et al. 2023 to reflect that it is now published. We have also amended the referencing algorithm to now display the websites for all online documents not published in peer-reviewed journals.

Reviewer #1

**General comments**

This is an excellent study and well-written manuscript. The paper will form an important contribution to the literature after very minor edits, which I outline below.

Thank you for this positive feedback.

**Specific feedback**

**Abstract**

- Third paragraph: *Fidelity to randomisation was impeded by strong cultural norms about what constituted IPV. However, the trial was feasible (recruitment rate: 1.5 per day; 37% of women screened were eligible).* Since randomization is a critical component of an RCT, it would be more accurate to state that recruitment (not the trial) was feasible.

We have amended this sentence as suggested:

However, recruitment ~~the trial~~ was feasible (recruitment rate: 1.5 per day; 37% of women screened were eligible).

- Likewise, it would make sense in paragraph four, regarding adjustments, to address the threat to randomization along with the other adjustments listed (e.g., training to identify psychological and emotional IPV).

We have amended this sentence to now read:

Adjustments to optimise a future, fully powered, randomised controlled trial include staggering recruitment in line with therapist availability, more training on the types of IPV and how to discuss~~ing IPV~~ them, automating randomisation, a supervision cascade model, and conducting post-intervention outcome assessments immediately and three months postpartum.

**Introduction**

- Excellent work identifying and justifying the population of focus (POF), the factors (and relationship between factors) affecting the POF’s health, the gaps in the lit, the logical intervention, and the rationale for a feasibility study. More information is needed, however, to explain why the authors believe a “brief” psychological intervention, as opposed to a more in-depth intervention, is an advantageous choice for this POF and setting. What lit corroborates this choice? What circumstances justify this choice? While I realize this was probably the subject of prior papers, please provide something here as well (including references to those prior, formative studies).

We have added the following paragraph (alongside sub-headings to guide readers through the Introduction):

Brief psychological interventions

Brief psychological interventions are condensed, simplified versions of talking therapies, which were often originally developed to be delivered over several months. Given healthcare resource constraints and very limited availability of mental health specialists in LMICs, the World Health Organization’s mental health gap action programme intervention guide (mhGAP-IG) recommends a range of evidence-based brief psychological interventions (12). An umbrella review of meta-analyses of studies conducted in low and middle-income countries (LMICs) identified 123 primary randomised controlled trials (RCTs; 13). Highly suggestive evidence of moderate credibility supported the efficacy of such brief psychosocial interventions in adults with CMDs in general settings (standardised mean difference=0.49, CI=0.36-0.62).

- I have only one other suggestion: First paragraph: *Incident depression was associated with intimate partner violence (IPV) during pregnancy.* I suggest including the applicable inferential stat estimate here, since OR is consistently reported above and would be informative. Also, please add citation for this sentence.

We have amended this sentence to now read:

Incident depression was associated with intimate partner violence (IPV) during pregnancy (adjusted risk ratio (aRR)=1.06, CI=1.00-1.12 (5)).

**Methods**

- Figure 1 is excellent. It is very easy to ascertain from the figure and the methods section text that bioethical requirements, including participant safety, were given foremost attention. The flow from recruitment through randomization into study arms is clear. The methods section provides a very informative level of detail. Missing, however, is whether the participants received compensation or other incentives for their participation.

This information was previously included in a paragraph underneath the ‘Masking’ sub-section. We have added an additional heading to make this information clearer, and moved the subsequent sentence about the support fund to the ‘Ethics and risk’ sub-section, as follows:

### Reimbursement

Participants were reimbursed for their time to attend baseline and outcome assessments, and for any transport costs incurred by participating in the study. All payment fees were set by the Addis Ababa University (AAU) CDT-Africa finance department, aligned with government compensation scales.

~~A fund was available to assist participants disclosing IPV, severe mental health symptoms, or suicidal ideation to access support services, such as transportation to a government social support office, police station, or secondary mental health service.~~

- Under “outcome measurement,” the authors state: *Outcome assessments were performed by masked, independent data collectors.* It was initially unclear to me if “masked” meant the assessors were blinded to participant assignment to study arm. This was clarified later. To avoid initial confusion, I suggest modifying the text to make this clear.

We have amended the text to now read:

Outcome assessments were performed by ~~masked,~~ independent data collectors blinded to participant allocation.

- Again, the methods section is comprehensive and clearly demonstrates the study’s rigor. This level of commitment to methodological detail is often missing in the lit, so I particularly appreciate the effort.

**Results**

- The results are comprehensive and articulated will in text and tables. An impressive amount of data were collected, and it is clear that the eventual RCT will likely capture sufficient data to measure direct in indirect effects. Obviously, measuring the statistical significance of pre-post differences was not applicable here. Nonetheless, the authors provide excellent summary tables that allow the reader to see how within and between subjects tests would be executed in a fully-powered RCT. Meanwhile, the study results are important and informative in their own right, addressing gaps the authors identified in the introduction. I have no changes to suggest here.

**Discussion**

- Paragraph three brings up important considerations regarding IPV disclosure. I agree with most of the logic. It would be interesting to stratify by IPV disclosure. However, disclosure would seem to play a powerful role in problem solving, as lack of disclosure is associated with avoidance coping. I am not suggesting changing the text, but it does appear to introduce coping style as a potential confounder.

We have added the following text to this section:

An American study found that avoidant coping mediated the association between negative social reactions to IPV disclosure and more severe PTSD symptoms.(74) A future RCT should therefore measure coping style as a potential confounder for any differences between the efficacy of problem-solving therapy in participants who do and do not disclose IPV exposure.

- Overall, the discussion is excellent. I have no suggestions for changes.

Reviewer #2

This article presents the feasibility testing of an adapted mental health intervention for postnatal women experiencing IPV. The article is well written and contributes important knowledge to the field of IPV intervention research. The article could be improved with some additional detail as noted in comments and questions below.

**Introduction**

- It would be good to make the case more clearly for why an intervention needs to be adapted to IPV and why PST is appropriate / evidence-based for this purpose

We have added the following text (alongside sub-headings to guide readers through the Introduction):

Trauma-informed care

Generic psychological interventions can neglect the role played by trauma in the mental ill-health of women experiencing IPV. Traumatic experiences (such as IPV) have lasting physical and psychological impacts, which influence a person’s relations to the world, self and others (14). People with lived experience of IPV have cautioned against ‘pathologising’ IPV-related difficulties, by considering mental health conditions in isolation, without addressing the emotional impact of IPV (15). Neglecting the wider needs of people experiencing IPV may reduce psychological interventions’ therapeutic potential (16). Trauma-informed care prioritises trauma awareness, safety, trustworthiness, choice, collaboration, strength, and skill-building (17).

Very few RCTs have evaluated psychological interventions for CMDs, tailored for women’s experiences of IPV in LMICs. Studies from Tanzania (18), Pakistan (19, 20), and Iran (21) support their potential benefits, but only one pilot RCT from Nepal focused specifically on pregnant women (22). We therefore conducted qualitative research (23), identifying a South African model of brief problem-solving therapy (PST; 24) as most contextually relevant to rural Ethiopia, and followed UK Medical Research Council (MRC)-affiliated ‘ADAPT’ guidance (25) to adapt it for the needs of pregnant women experiencing IPV in this setting.

**Methods**

- Rigorous use of ADAPT framework and following MRC guidance on developing interventions prior to testing, as well as a strong 3-arm design. Appreciate the testing of IPV-adapted vs. normal PST.
- Specify what happened for women who screened out due to illness or need for treatment

We have added the following sentence to the text about exclusion criteria:

Any women assessed as being at imminent risk from physical or mental ill-health were referred to the primary hospital, for escalation of their care.

- Can you clarify that the ‘sources of support’ provided to the EUC included mental health and GBV service providers that were broadly accessible in terms of cost, location?

We have amended the text to now read:

EUC comprised a leaflet on well-being during pregnancy and information about free, local sources of support available to women and families, provided by the field supervisor at the time of randomisation. As no specific perinatal mental health or IPV services were available at the time of the study, sources of support constituted general provision such as the government-funded Women and Child Affairs Office.

- No socioeconomic indicators were collected? Food security?

Upon careful consideration, we omitted food insecurity from baseline data collection due to the already lengthy battery of assessments being collected.

Outcomes

- Did you use any frameworks to inform your definitions of feasibility and other outcomes?

We have amended the text to now read:

We operationalised each of these outcomes as follows, informed by the definitions in Proctor and colleagues’ taxonomy (36):

- Why is recruitment rate also a measure of appropriateness, in addition to feasibility? How do you distinguish its role in each of these?

We judged that recruitment rate indicated both the practical ability to recruit the sample size required for the study design and the relevance/appropriateness of the study design to the context. We have amended the text to now read:

- Feasibility: recruitment rate (feasibility of recruiting the required sample size), outcome assessment completion, supervision attendance…
- Appropriateness: recruitment rate (numbers of eligible women identified who consented to take part).

Theory of change and mediators

- Appreciate the use of a theory of change and measurement of mediators. Did you consider acceptability of violence?

Several items in the WHO Attitudes Towards Gender roles instrument explore this point, such as “In your opinion, does a man have good reason to hit his wife if: (a) she does not complete her household work to his satisfaction, (b) she disobeys him, (c) she refuses to have sexual relations with him, (d) she asks him whether he has other girlfriends, (e) he suspects that she is unfaithful, (f) he finds out that she has been unfaithful?”

We did not analyse individual items on the instrument due to the small sample size but we would do so in a fully powered RCT.

- In `terms of healthcare-seeking behavior, did it include seeking non-clinical services that may also be supportive for someone experience violence, e.g. legal, housing, etc.? Specify.

We have amended the text to now read:

Finally, we measured healthcare-seeking behaviour (including from non-biomedical sources such as religious healers, herbalists, and holy water sites), using the Client Service Receipt Inventory (CSRI) (64), adapted for Ethiopia (65) and modified to focus on the past three months, except for inpatient care at baseline, which was asked for the past 12 months.

CSRI does not include non-healthcare help seeking but additional items about violence-relevant help-seeking could be interesting to measure in an RCT.

Training

- Specify that the different sets of trainers was to limit contamination, if it was (p. 11)

We have done so:

To limit contamination between arms, a separate group of six ANC providers received standard PST training, delivered by a different psychiatrist and psychologist to PST-IPV, using the same five day structure.

Randomization

- I’m a little confused about how this is described. Were all women reporting IPV randomized to IPV-PST? How is that so since eligibility included reporting IPV?

We have amended this description to now read:

A three-arm randomisation table was generated by the trial statistician (who was not otherwise involved in data collection processes) using a random number list. The table proportionately allocated ~~eligible~~ women eligible for this trial (experiencing depressive symptoms and IPV) in the ratio of 2:1:1 to PST-IPV, standard PST, and EUC arms.

The separate, nested feasibility trial used a different ~~second~~ (two-arm) randomisation table to allocate women experiencing depressive symptoms but not reporting IPV in a 1:1 ratio, to standard PST and EUC arms.

~~Eligible~~ Women eligible to take part in either of the two trials were randomised using these tables until 25 women had been randomised to an arm, at which point enrolment into that arm was closed and recruitment continued for the remaining, unfilled arms.

Protocol deviations

- Thank you for reporting this important deviation and the likely very common misclassification of GBV

**Results**

- Did you look at whether characteristics of those who did not complete follow up were the same as those who did? I know it’s a small sample so looking for significant differences wouldn’t be meaningful, but from a descriptive perspective it could be interesting to know

Thank you for this suggestion. We have examined this question and added the following text to the Results:

Of the 13 participants out of 52 (25%) not completing outcome assessments, five (38%) moved away, four (31%) could not be traced, and four declined, usually due to a cultural practice of postnatal confinement. The median age of women who did not complete outcome assessments (n=13) was 20 years (IQR=18-26) in comparison to 25.5 years (IQR=24-30) for those (n=39) who did. Baseline IPV scores were similar (median NGL score was 4 in both groups, median WHO IPV score was 5 (IQR=1-11) in the follow-up non-completion group and 6 (IQR=3-10) in the completion group). Due to the small number of participants who did not complete the outcome assessment, we did not perform statistical comparisons. However, baseline trauma symptoms may have been higher in the group not completing outcome assessment (median PCL-5=9, IQR=6-13) than in the group which did (PCL-5=6, IQR=3-12). Baseline differences in anxiety (median GAD-7=11 (IQR=9-12) compared to 10 (IQR=7-12)) and depressive symptoms (median PHQ-9=9 (IQR=8-10) compared to 8 (IQR=7-11)) were less pronounced.

We have also added the following text to the Discussion:

Home visits for outcome assessment could also be explored but preserving confidentiality and women’s safety should remain paramount. The small group which did not complete outcome assessments appeared to be younger than the group which did, highlighting the importance of optimising intervention design and trial procedures for groups known to be especially vulnerable to IPV (93). The fact that women who did not complete outcome assessments appeared to have higher trauma symptoms than those who did is relevant to the finding that sessions did not focus on IPV and the possibility of avoidant coping.

Acceptability

- How did you assess if participants comprehended randomization? (p. 19)

We have amended this point in the Methods section as follows:

Acceptability: uptake of initial screening, proportion of eligible women participating, comprehension and acceptance of randomisation (recorded in field journals by research staff screening women and inviting them to participate), ANC provider drop-out rate, proportion completing four sessions, acceptance of audio-recording.

- Was staff drop out the only measure of their acceptability?

We have added the following text to the limitations section:

This study only reports quantitative feasibility measures. A future study will analyse qualitative interviews with pregnant women who received problem-solving therapy and ANC providers who delivered it, to explore these constructs in depth.

Appropriateness

- How did you determine a cut-off of 30 min in terms of appropriateness of integration into ANC?

We apologise for our lack of clarity. We have rephrased this point as follows:

Table 2 shows that the mean ~~session~~ duration~~s~~ of each session ranged from 32~~over 30~~ minutes (~~for all~~ session 4~~s~~) to 51 minutes (session 2). This finding raised the possibility that ~~the problem-solving therapy model may not have been appropriate for integration into ANC, as~~ the content which ANC providers were instructed to cover within ~~per~~ each session ~~may have been~~ was excessive and so the intervention model may not have been appropriate for integration into ANC.

Fidelity

- Very important and useful findings around the issues with fidelity, these are common and therefore important to highlight for others, including the avoidance of the topic of violence among providers, interesting though that the quality scores were still quite high in Table 3, as were responsiveness. Can you comment on that in the discussion?

We have added the following text to the Discussion:

Most women completed all four intervention sessions. The acceptability of PST-IPV may have arisen from the very limited opportunities women in this context have to be listened to, encouraged, and emotionally supported: the sixth international standard of maternal and new-born healthcare (75). Uncompassionate and disrespectful maternity care has been reported in LMICs including Ethiopia, especially towards women in rural areas (76). Caring, respectful and compassionate (CRC) care has been prioritised (77), with a national transformation agenda for a motivated, competent and compassionate health workforce (78). In our study, mental health specialists supervising ANC providers recommended supplementary communication skills training but independent ratings of session quality and therapist responsiveness were high, supporting the feasibility of training non-specialist staff to deliver a simplified psychological intervention in this context.

However, we found that IPV was not the primary focus of PST-IPV sessions…

Safety

- What were the mechanisms of tracking and documenting safety outcomes?

We have added the following text to the Methods section:

Safety: frequency of physical health, mental health, and safety-related adverse events. As previous research in this setting found that perinatal complications were not reliably self-reported (53), births, stillbirths, and early neonatal deaths were collected from HEWs, who maintain maternal care records. Any mental health and safety concerns identified during the study were discussed with the project mental health professional, who kept a log and followed up adverse events.

Table 4

- Why did you ask about past year IPV at follow-up when it hadn’t been a year since baseline?

We had planned to ask about past-month IPV at follow up but past year was asked about instead. We have amended the text to now read:

Reported IPV reduced across all arms, post-participation but interpretation was complicated by the time periods asked about overlapping. An RCT of the common elements treatment approach delivered by lay workers in Zambia found significant reductions in self-reported IPV exposure at one (106) and two years’ follow-up (107). Qualitative interviews (108) identified safety strategies to avoid or prevent conflict and control anger, improved trust and understanding, as mediators. A future RCT should measure hypothesised interpersonal mediators of change, as well as psychological constructs, such as self-efficacy and ensure that follow-up questions about IPV specify a shorter time period.

- And what was the time period used for the WHO questions? Include that information in the measures section where it would also be useful to list some example items.

We have added the following text to the description of the IPV measures:

An example item on the non-graphic language screen is “how is your partner treating you (and your children)?” (Always well/well most of the time/neutral/not well most of the time/never well). An example item on the WHO instrument is “the next questions are about things that happen to many women, and that your husband may have done to you. I want you to tell me if your husband or any other partner has ever done the following things to you in the past 12 months. (a) Insulted you or made you feel bad about yourself? (Yes/No. If yes, how many times in the past 12 months)?”

- It would also be helpful to describe the range for ‘attitudes towards gender roles’ and interpretation in the table footnotes; same for other outcome measures for ease of interpretation

We have amended the footnotes for Table 1 as follows:

…LTE: list of threatening experiences (possible scores: 12 (most stressful life events) – 24 (fewest stressful life events))…

In Table 4 we have now added the possible score range in the first column, for ease of interpretation.

**Discussion**

- You raise important points in the discussion, including whether IPV should be an eligibility criteria in an area of high exposure, which I think it may not need to be and your proposal for a stratified analysis is useful.
- In terms of discussing IPV during the counselling sessions, you suggest women may not want to talk about the IPV and rather other stressors they face. Do you however have data categorizing the types of other stressors discussed by women? I imagine from the recordings you could assess that, but also glean if it was more provider avoidance than women’s desires per se. I think some more information around that would be useful and/or useful to explore in follow-on in-depth interviews with women and providers. My hunch is it was more discomfort as in my experience, women are highly interested in exploring relationship challenges faced, which intersect with other socio-economic challenges in their lives.

We are planning to study this question. We have added the following text:

Future research could evaluate the content of intervention sessions to quantify time spent discussing different types of problems and clarify whether providers, participants or both actively avoided talking about IPV.
